# Editorial: Halal production, services, consumption, and consumer behavior

**DOI:** 10.3389/fpsyg.2022.1104099

**Published:** 2023-01-05

**Authors:** Abdul Hafaz Ngah, T. Ramayah, Mohammad Iranmanesh, Suhaiza Zailani

**Affiliations:** ^1^Marketing Department, Faculty of Business, Economics and Social Development, Universiti Malaysia Terengganu, Kuala Terengganu, Malaysia; ^2^School of Management, Universiti Sains Malaysia (USM), George Town, Malaysia; ^3^Department of Information Technology & Management, Daffodil International University, Dhaka, Bangladesh; ^4^Fakulti Ekonomi dan Pengurusan (FEP), Universiti Kebangsaan Malaysia (UKM), Bangi, Malaysia; ^5^Azman Hashim International Business School, Universiti Teknologi Malaysia (UTM), Johor Bahru, Malaysia; ^6^Applied Science Private University (ASU), Amman, Jordan; ^7^University Center for Research & Development (UCRD), Chandigarh University (CU), Sahibzada Ajit Singh Nagar, Punjab, India; ^8^School of Business and Law, Edith Cowan University, Joondalup, WA, Australia; ^9^Faculty of Business and Accountancy, Universiti Malaya, Kuala Lumpur, Malaysia

**Keywords:** systematic literature review, future behaviors, religiosity, halal consumer behavior, Smart Partial Least Squares (PLS)

## 1. Introduction

The Muslim population is projected to reach 2.2 billion by the year 2030. The rapid growth of the Muslim population will positively impact the demand for halal products and services, as Muslims should only consume halal products. However, due to high quality and purity, Halal products and services were only limited to Muslims, but more recently the non-Muslims have also started to enjoy its benefits. As a result, a growing number of producers and manufacturers, including non-Muslim ones, have begun to produce Halal products and deliver Halal services. Previously, Halal was only related to food and beverages. Nowadays, the Halal industry has expanded its market into a wide range of products and services such as cosmetics, pharmaceuticals and personal care, supply chain, and banking services. Products fall into three categories according to their halal status: certified halal products, non-certified halal products, and non-halal products. Despite many studies conducted on Halal products, the role of psychological factors has received less attention. Consumer choices and behaviors are influenced by various psychosocial factors. Many studies have been conducted on Halal either at the organizational level (Ngah et al., 2021a) or individual level (Iranmanesh et al., 2019). The studies have investigated the Halal concept in various products and services such as supply chain (Fathi et al., 2016; Zailani et al., 2017; Ab Talib et al., 2020; Ngah et al., 2021a), food, and beverages (Zailani et al., 2019; Halimi et al., 2021), cosmetics (Suparno, 2020; Ngah et al., 2021b), and pharmaceutical (Ngah et al., 2019; Widyanto and Sitohang, 2021). The halal concept continues to receive considerable attention from researchers.

## 2. Aims of the special issue

In light of the above, this special issue focuses on the role of psychological factors in the Halal context aiming to enable managers to develop better marketing strategies, opportunities, and innovative solutions.

### 2.1. Review of the contributions

We received a total of 11 submissions. After careful screening, six papers were rejected either due to being out of scope, or the manuscripts have not met the standard requirements of this reputable journal. Following the journal's guidelines, the rest of the manuscripts were sent for peer review to two or more experts in the area of studies. After a few rounds of revisions, five articles were accepted for this issue. The first paper by Dewi et al. discusses the halal literature from Indonesia and Spain. The next paper by Tao et al. investigates the unwillingness to buy halal-made products among Indian consumers. The third article by Huang et al. investigates the negative effects of customer bullying among frontline employees in the Chinese hotel industry and food catering industry. The next article by Jiang et al. assesses the new marketing strategies for online group buying in China. The last article by Ai et al., unearths the issue of patients' satisfaction and trust toward healthcare service environments from the perspectives of patients in Malaysia. Twenty authors were involved in writing these five articles, seven authors from China, five from Indonesia, three from Malaysia, and one author from Spain, Jordan, India, Bangladesh and the United states of America.

## 3. Concluding remarks

The special issue contributes to the halal literature and provides practical insights into Muslim and non-Muslim customer behaviors for managers in the halal industry. The published articles are not only beneficial for academicians but also provide valuable information for industry players and policymakers.

## Author contributions

All authors listed have made a substantial, direct, and intellectual contribution to the work and approved it for publication.
